# Higher Brain Glutathione Levels Relate to Better Cognitive Performance in Older Adults

**DOI:** 10.1002/alz70856_106151

**Published:** 2026-01-08

**Authors:** Phil Lee, Kirk I. Erickson, Chaeryon Kang, Jeffrey M. Burns, Charles Hillman, Arthur F. Kramer, George Grove, Eric D Vidoni, Haiqing Huang, Edward McAuley, Lu Wan, Lauren Oberlin, In‐Young Choi

**Affiliations:** ^1^ University of Kansas Medical Center, Kansas City, KS, USA; ^2^ AdventHealth Research Institute, Orlando, FL, USA; ^3^ University of Pittsburgh, Pittsburgh, PA, USA; ^4^ Northeastern University, Boston, MA, USA; ^5^ Beckman Institute, University of Illinois, Urbana, IL, USA; ^6^ Center for Cognitive & Brain Health, Northeastern University, Boston, MA, USA; ^7^ AdventHealth Research Institute, Neuroscience, Orlando, FL, USA; ^8^ University of Illinois, Urbana, IL, USA; ^9^ AdventHealth Neuroscience Institute, Orlando, FL, USA

## Abstract

**Background:**

The free radical theory of aging posits that oxidative stress is a key mechanism underlying aging and the onset and progression of neurodegeneration, leading to dementia and Alzheimer's disease (AD). Brain tissue undergoing oxidative stress is reflected by lower levels of brain glutathione (GSH), the major antioxidant in the brain, which provides a first line of defense against free radicals that are known to cause cellular damage, impaired cell function and eventual cell death. Brain GSH levels are depleted in individuals with mild cognitive impairment and AD, as GSH is consumed in this protective process. GSH levels are also tied to AD neuropathology, amyloid‐β and tau, as well as with bioenergetics and mitochondrial function, all features underscoring aging and dementia. However, there remains a poor understanding of how GSH relates to different cognitive domains, especially in cognitively unimpaired older adults. We predicted that higher levels of GSH would reflect elevated antioxidant defenses and relate to better cognitive function.

**Method:**

We employed MR spectroscopy (MRS) with advanced multiple quantum chemical shift imaging technique to map GSH levels in the human brain. Older adults (*n* = 206, 63 M, 143 F; age 69.8±3.9 years) from three geographical regions (Kansas City, Pittsburgh, Boston) underwent MR scans and a battery of cognitive testing for working memory, episodic memory, visuospatial processing, attentional control, and processing speed. A multiple regression model was used to evaluate the association between brain GSH and cognitive performance in each domain.

**Result:**

Brain GSH levels were correlated positively with working memory (*p* = 0.019), episodic memory (*p* = 0.030), and visuospatial processing (*p* = 0.003), adjusted for sex, age and years of education. However, neither attentional control (*p* = 0.54) nor processing speed (*p* = 0.90) were significantly associated with brain GSH levels.

**Conclusion:**

Overall higher brain GSH levels were linked to better episodic memory, working memory, and visuospatial performance in older adults. Our findings contribute to our understanding of the potential role of antioxidant defenses in brain aging, suggesting that enhancing brain GSH levels may constitute a route for improving cognitive function in older adults.

